# Nonthrombotic Pulmonary Embolism: A Potential Complication of Polyacrylamide Hydrogel Cosmetic Injection

**DOI:** 10.1155/2016/1397434

**Published:** 2016-01-14

**Authors:** Faisal Inayat, Ahmad R. Cheema, Hafeez Ul Hasan Virk, Daniel J. Yoon, Salman Farooq, Abdul Manan

**Affiliations:** ^1^Department of Medicine, New York-Presbyterian Hospital, Weill Cornell Medical College, New York, NY 10065, USA; ^2^Department of Medicine, Mount Sinai St. Luke's and Mount Sinai Roosevelt Hospitals, Icahn School of Medicine, New York, NY 10019, USA; ^3^Department of Radiology, Mount Sinai St. Luke's and Mount Sinai Roosevelt Hospitals, Icahn School of Medicine, New York, NY 10025, USA; ^4^Department of Neurology, Froedtert Memorial Lutheran Hospital, Medical College of Wisconsin, Milwaukee, WI 53226, USA

## Abstract

*Context.* Polyacrylamide hydrogel (PAAG) has gained importance as a synthetic soft tissue filling agent. It has been commonly employed by physicians in Europe for facial contouring and soft tissue augmentation. Previously, PAAG is considered nontoxic and well tolerated with a few mild procedural complications.* Case Presentation.* A 26-year-old female was hospitalized for dry cough, worsening dyspnea, and chest discomfort after 3 hours of multiple PAAG injections in buttocks. The patient's condition deteriorated and rapidly advanced to acute respiratory failure. Therein, the diagnosis of nonthrombotic pulmonary embolism (NTPE) was established on standard set of investigations. She was intubated; corticosteroid and empiric antibiotic therapy was initiated resulting in improvement of her condition. Subsequently, extubation was done, and she was discharged from the hospital after an uneventful recovery. On 1-month follow-up, the patient had no previous symptoms.* Conclusion.* This report implicates clinicians to maintain a high index of suspicion for NTPE in patients presenting with respiratory symptoms following PAAG usage.

## 1. Introduction

Injectable fillers are one of the popular nonsurgical treatments for wrinkles and facial contouring in Europe. One such filler is polyacrylamide hydrogel (PAAG), which is currently approved in various countries for breast augmentation, facial contouring, and correction of HIV-associated facial lipoatrophy, except the United States. Several adverse effects of PAAG have been reported in the literature, including local infection, inflammation, pain, nodule formation, and delayed hypersensitivity reaction. However, we report a rare complication in the form of severe nonthrombotic pulmonary embolism (NTPE) after 3 hours of PAAG administration for buttock augmentation in a 26-year-old female. The patient was managed with intubation, corticosteroids, and antibiotics resulting in diminution of symptoms. Subsequently, the patient was extubated and was discharged from the hospital. She has been disease-free since then.

## 2. Case Presentation

A 26-year-old female presented to the Emergency Department of Mount Sinai St. Luke's and Mount Sinai Roosevelt Hospitals with gradually worsening shortness of breath, dry cough, and substernal chest discomfort for 3 hours. She had bilateral multiple injections of polyacrylamide hydrogel (PAAG) (Aquamid) in the buttocks for cosmetic enhancement. The patient reportedly remained stable during the procedure. However, cough, chest discomfort, and dyspnea developed after 1 hour, which gradually worsened, and she presented to the emergency department 3 hours after the procedure.

On admission, her vital signs revealed body temperature of 37.7°C, blood pressure of 110/74 mmHg, pulse rate of 84 beats/min, and respiratory rate of 42/min. The patient was tachypneic with arterial oxygen saturation of 88% on room air. She appeared confused on mental status examination. On chest auscultation, crackles were audible in both the lower lung fields. Multiple stigmata of recent injections were seen on buttocks bilaterally.

**Figure 1:**
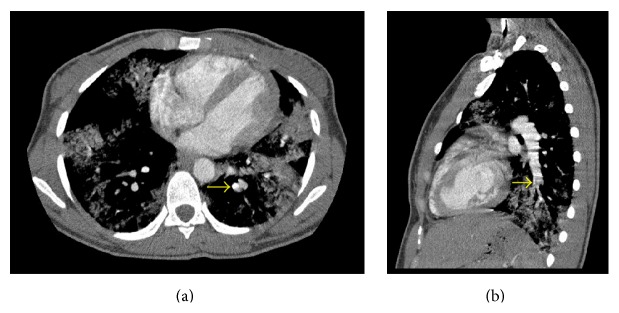
Volumetric computed tomography acquisition of the chest from the thoracic inlet to the upper abdomen, following 65 mL of intravenous contrast (Omnipaque 300) employing pulmonary embolism protocol. A filling defect has been demonstrated in the left lower lobe segmental pulmonary artery (arrows: (a) axial; (b) sagittal).

**Figure 2:**
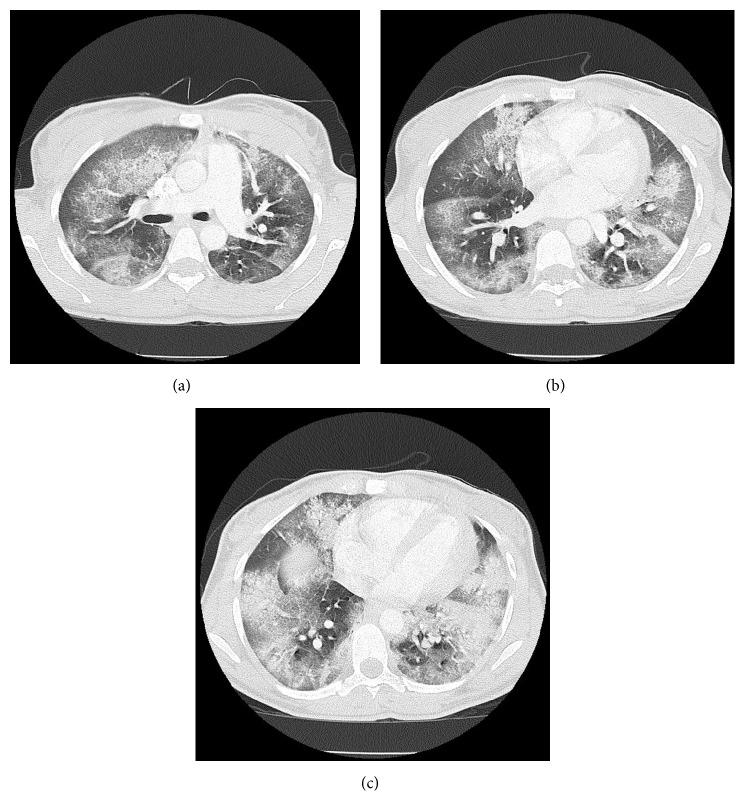
Contrast-enhanced computed tomography demonstrating extensive diffuse ground-glass opacifications, with more confluent opacity involving the lower lobes.

**Figure 3:**
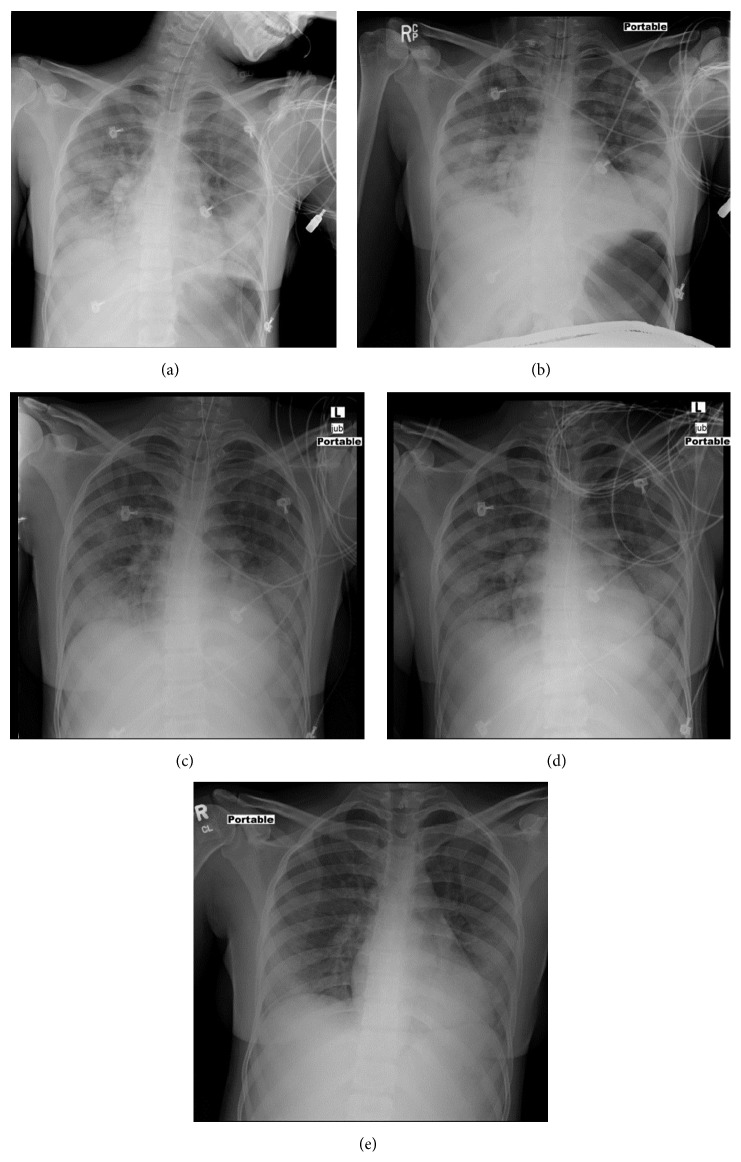
(a)–(d) Endotracheal tube is noted with the tip ending at the satisfactory position. An enteric tube has been placed. Diffuse bilateral hazy opacification is again seen, which gradually improved in sequential (a)–(d) chest radiographs. (e) Chest radiograph showing marked improvement in previously seen bilateral hazy opacities after extubation.

Initial laboratory evaluation revealed the following: white cell count, 3.7 K/*μ*L (3.8–9.8); hemoglobin, 12.3 g/dL (11.6–15.3); platelets, 211 K/*μ*L (150–450); troponin I, 0.013 ng/mL (0–0.034); CK-MB, 0.9 ng/mL (0–3.38); C-reactive protein, 6.412 mg/dL (0–0.5); D-dimer, 1.37 *μ*g/mL (0–0.5); and B-type natriuretic peptide, 9.0 pg/mL (0–100). Complete hepatic panel and renal function tests were normal. Arterial blood gas analysis was as follows: pH, 7.42; PCO_2_, 38.4 mmHg; PO_2_, 72.6 mmHg; bicarbonate, 24.1 mmol/L. Electrocardiogram showed a right bundle branch block (RBBB) pattern. In the ED, the patient went into acute hypoxic respiratory failure and an intubation tube was immediately passed to maintain the airways. Volumetric computed tomography (CT) showed a filling defect in the left lower lobe segmental pulmonary artery (Figures 1(a) and 1(b)). In addition, there were extensive diffuse ground-glass opacifications, with more confluent opacity, involving the lower lobes (Figures 2(a)–2(c)). Endotracheal tube was noted with the tip ending at the satisfactory position. An enteric tube had been placed. Diffuse bilateral hazy opacity had been seen again, which gradually improved in sequential radiographs (Figures 3(a)–3(d)).

**Figure 4:**
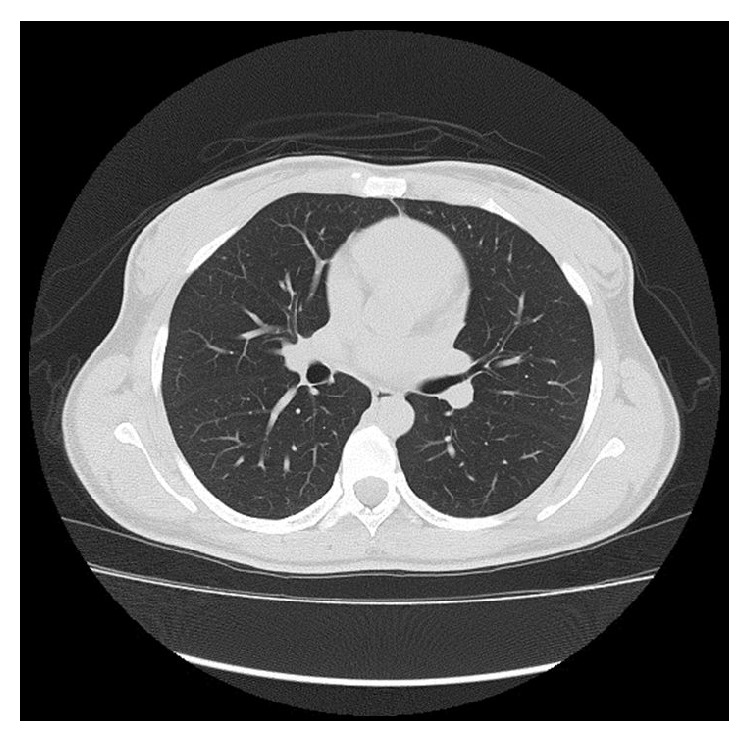
Contrast-enhanced computed tomography on 1-month follow-up demonstrating complete resolution of the parenchymal opacifications.

On bronchoscopic examination of inner airways, diffuse alveolar hemorrhages were revealed with no overt source of bleeding. Bronchoalveolar lavage (BAL) fluid cytology was positive for alveolar macrophages and mixed inflammatory cells. Work-up for connective tissue and autoimmune diseases demonstrated negative results. Hence, the diagnosis of nonthrombotic pulmonary embolism (NTPE) was confirmed on the basis of history taking, physical exam findings, and radiologic and pathologic investigations. The treatment in our patient was started with corticosteroids and empiric antibiotics. Subsequently, a marked improvement in previously seen bilateral hazy opacities was demonstrated (Figure 3(e)). She was eventually extubated. The patient initially reported a little difficulty in breathing which resolved in 24 hours. She was discharged from the hospital after an unremarkable recovery. Follow-up CT after a month demonstrated complete resolution of the parenchymal opacifications (Figure 4).

## 3. Discussion

Polyacrylamide has been considered as a nontoxic polymer constituted by acrylamide subunits. It is highly water-absorbent and results in polyacrylamide hydrogel (PAAG) on hydration. PAAG contains 2.5% polyacrylamide and 97.5% water for injection purpose [1]. In 1980, it demonstrated good clinical outcomes as permanent orthopedic filler in Ukraine [2]. Europe and a few other countries legalized its use for facial contouring, breast, and soft tissue augmentation [3, 4]. However, it has not been approved by Food and Drug Administration (FDA) in the United States. Despite the fact that PAAG usage has not been recommended in formal medical institutions, illegal procedures employing PAAG are performed in many clinics and beauty parlors across the United States. People tend to use PAAG for cosmetic reasons due to low cost and rapid, but long-lasting results. This case illustrates the same practice culminating in an unusual and serious complication.

Patients following PAAG toxicity usually present with nonspecific, subchronic, and cumulative symptoms. They predominantly include the following: numbness, ataxia, and skeletal muscle weakness in limbs; hyperhidrosis of hands; erythema and peeling of the palms; and generalized lethargy [5–7]. Most notable early complications are focal lumps, inflammation, infection, asymmetry, hematomas, irregularity of the injection site, keloid formation, gel migration, pain in cold weather and feeling of expansion in warm environment, skin necrosis, and hyperpigmentation [1, 8–10]. In newer studies, PAAG has been associated with neurotoxicity, genotoxicity, carcinogenicity, mutagenicity, and reproductive toxicity [11–14]. However, nonthrombotic pulmonary embolism (NTPE) secondary to PAAG injection is a rare clinicopathologic entity. To our research, there is only one case of PAAG-induced NTPE in the literature [15].

NTPE is an uncommon, underdiagnosed, and life-threatening condition. It frequently presents with unusual and vague clinical manifestations, which can be dramatically acute or overwhelmingly delayed. It indicates the need for detailed history taking and thorough physical examination. Furthermore, laboratory, radiologic, and pathologic investigations hold particular importance to reach an accurate diagnosis [16, 17]. Radiologic manifestations such as lung opacities and indications of embolism prompt physicians to seek pathologic analysis. Pathological findings from bronchoalveolar lavage (BAL) and lung biopsy are often confirmatory. However, the problem arises when a clinician does not consider a computed tomography (CT) scan for the potential NTPE case, partly due to mild presenting symptoms or lack of knowledge on this condition. On this basis, NTPE has been a formidable diagnostic challenge. The missed diagnoses in patients with NTPE have been associated with significantly high morbidity and mortality. Clinicians need to be particularly vigilant when a patient presents with respiratory symptoms after PAAG usage.

NTPE can be caused by a wide range of nonthrombotic sources, which include different cell types (adipocytes, haematopoietic, amniotic, trophoblastic, or tumor), bacteria, fungi, foreign materials, or gas [18]. The pathophysiology of cosmetic filler-induced NTPE includes high-pressure injection, large filler volume injection, massage or trauma at the injection site, and direct injection into a vein [19, 20]. Furthermore, migration of implant following the injection of PAAG in areas with extensive movement such as buttocks may have a role in development of NTPE. In our patient, the interval between PAAG administration and manifestation of symptoms was only 3 hours suggesting likely systemic embolization.

## 4. Conclusion

PAAG injections, as in our case, may lead to serious and life-threatening nonthrombotic pulmonary embolism. Low specificity of the clinical manifestations makes the clinical diagnosis difficult. Knowledge and awareness of radiologic and pathologic findings associated with nonthrombotic pulmonary emboli are essential to reach a correct diagnosis. Embolism of injected material should always be in the differential diagnoses in patients developing acute respiratory failure after cosmetic procedures.
